# Conditional probabilistic diffusion model driven synthetic radiogenomic applications in breast cancer

**DOI:** 10.1371/journal.pcbi.1012490

**Published:** 2024-10-07

**Authors:** Lianghong Chen, Zi Huai Huang, Yan Sun, Mike Domaratzki, Qian Liu, Pingzhao Hu

**Affiliations:** 1 Department of Computer Science, Western University, London, Ontario, Canada; 2 Department of Biochemistry, Schulich School of Medicine & Dentistry, Western University, London, Ontario, Canada; 3 Department of Applied Computer Science, University of Winnipeg, Winnipeg, Manitoba, Canada; 4 Department of Epidemiology and Biostatistics, Schulich School of Medicine & Dentistry, Western University, London, Ontario, Canada; 5 Department of Oncology, Schulich School of Medicine & Dentistry, Western University, London, Ontario, Canada; 6 The Children’s Health Research Institute, Lawson Health Research Institute, London, Ontario, Canada; Georgia Institute of Technology, UNITED STATES OF AMERICA

## Abstract

This study addresses the heterogeneity of Breast Cancer (BC) by employing a Conditional Probabilistic Diffusion Model (CPDM) to synthesize Magnetic Resonance Images (MRIs) based on multi-omic data, including gene expression, copy number variation, and DNA methylation. The lack of paired medical images and genomics data in previous studies presented a challenge, which the CPDM aims to overcome. The well-trained CPDM successfully generated synthetic MRIs for 726 TCGA-BRCA patients, who lacked actual MRIs, using their multi-omic profiles. Evaluation metrics such as Frechet’s Inception Distance (FID), Mean Square Error (MSE), and Structural Similarity Index Measure (SSIM) demonstrated the CPDM’s effectiveness, with an FID of 2.02, an MSE of 0.02, and an SSIM of 0.59 based on the 15-fold cross-validation. The synthetic MRIs were used to predict clinical attributes, achieving an Area Under the Receiver-Operating-Characteristic curve (AUROC) of 0.82 and an Area Under the Precision-Recall Curve (AUPRC) of 0.84 for predicting ER+/HER2+ subtypes. Additionally, the MRIs served to accurately predicted BC patient survival with a Concordance-index (C-index) score of 0.88, outperforming other baseline models. This research demonstrates the potential of CPDMs in generating MRIs based on BC patients’ genomic profiles, offering valuable insights for radiogenomic research and advancements in precision medicine. The study provides a novel approach to understanding BC heterogeneity for early detection and personalized treatment.

## Author summary

Breast cancer (BC) is known for its diverse characteristics, which makes it crucial for early detection and personalized treatment. Combining medical images with genomics provides a fresh approach to studying this diversity, leading to the emergence of a new field called radiogenomics.

However, when these two data types (image data and genomic data) are not paired, it becomes a challenge. This study proposes the use of a well-trained Conditional Probabilistic Diffusion Model (CPDM) to address this issue by generating BC medical images based on genomic information. CPDM is a type of advanced Artificial Intelligence (AI)-based generative model, like ChatGPT. CPDM has been very successful in creating images that look real. In this study, we built and trained a CPDM specifically for BC. The well-trained CPDM can generate BC medical images very well and the generated images can accurately predict patients’ clinical attributes like gene mutations, receptor statuses, and survival probabilities. This research explores the potential of using CPDMs to generate meaningful medical images from genomic data, aiding in solving crucial clinical problems. These findings have implications for advancing radiogenomic research and the development of personalized medicine approaches using AI.

## 1. Introduction

In the recent years, especially during the COVID-19 pandemic, a significant number of Breast Cancer (BC) patients missed the optimal opportunity for cancer diagnosis and treatment [1]. Additionally, the virus infection might lead to the awakening of dormant BC cells in the bodies of some otherwise healthy people [2]. These factors have contributed to an increase in the number of new and fatal cases of BC. A survey conducted in 2023 revealed that 353,510 women in the United States would be diagnosed with BC and 43,170 women were anticipated to die from BC [3–4]. Therefore, how to diagnose BC early and improve the survival rate of BC patients has become an important topic. However, the heterogeneity of BC posed significant challenges for its early detection and treatment. Specifically, genetic, molecular, and cellular variations within and between tumors could lead to different subtypes of BC, responding variably to the same treatment [5]. Nonetheless, traditional methods struggled to detect the heterogeneity of BCs effectively. Radiogenomics study is a promising direction in this field.

BC radiogenomics study focused on the relationship between imaging phenotypes and genomics [6]. A previous study showed that radiogenomic analysis could reveal voxel-by-voxel genetic information of a heterogeneous tumour, which could guide personal treatment [7]. In addition, radiogenomics could quantify lesion characteristics to distinguish benign and malignant entities as early as possible, allowing physicians to better stratify patients according to disease risk and perform more precise imaging and screening [7]. However, a conventional radiogenomic study typically demanded medical image data, genomics data, and clinical data to be collected from the same cohort, which was usually not achievable. Recently, with the development of deep generative models such as ChatGPT, researchers were able to synthesize images from other information. Investigation has shown that deep generative models have good performances in the synthesis of medical images of the brain, liver, lung, and other organs [8–12]. Currently, there is no study that generates synthetic medical images for BC radiogenomic analysis.

There were many classical generative models in the deep learning field, such as AutoEncoder (AE), Variational AutoEncoder (VAE), Transformer, Generative Adversarial Network (GAN), and so on. However, these models demonstrated certain limitations in past research. AE and VAE models may often produce unrealistic and fuzzy samples [13]. Transformers were generally resource-intensive, requiring significant computational power and memory. Transformers alone tend to face difficulties in producing satisfactory outcomes, particularly when data is scarce or costly to obtain [14]. GAN models were known for their potential instability during training, challenging convergence, and limited diversity in generating outcomes [15].

Recent diffusion models in deep learning offer a promising approach to address some of the limitations commonly faced by traditional deep learning models, particularly in scenarios of high-quality and diverse sample generation. The diffusion model was conceptually inspired by the stochastic diffusion process found in non-equilibrium thermodynamics [16]. It defined a Markov chain to add random noise to samples step-by-step, and then learned to reverse the noise-adding process by a deep learning model to generate new samples [16]. This characteristic enabled diffusion models to intricately construct complex details in the generated samples, circumventing common pitfalls such as mode collapse often encountered in traditional generative models, thereby ensuring a more stable and less adversarial training process [16]. The strengths of diffusion models facilitated their broad utilization in diverse fields. The well-applied diffusion models included the probabilistic diffusion model, Denoising Probabilistic Diffusion Model (DDPM), DALLE, stable diffusion model [17–20], etc. Nonetheless, to the best of our knowledge, generating high-quality BC MRIs utilizing diffusion models according to patients’ genomics profiles was still an unexplored research field.

In addition to synthesizing missing imaging data, predicting clinical attributes including mutations in BC driver genes, Estrogen Receptor (ER) status, ER-positive/Human Epidermal growth factor Receptor 2-positive (ER+/HER2+) subtypes, prognosis, and treatment efficacy based on MRIs also played an important role in BC radiogenomic studies [21–24]. Specifically, scientists could design personal treatment for individuals according to the BC driver gene mutation status of patients. Moreover, for patients with ER+ cancer, given the hormone sensitivity of their cancer cells, physicians could employ targeted hormone therapy to enhance treatment efficacy while minimizing potential harm to normal cells. For patients with ER+/HER2+ subtypes of BC, targeted therapies that specifically inhibited HER2 receptors and modulated estrogen effects could significantly improve treatment outcomes. Survival analysis in BC patients allowed for a more nuanced understanding of prognosis, enabling healthcare providers to identify high-risk individuals and optimize treatment strategies to extend survival and improve quality of life. However, obtaining these clinical attributes in practice often requires invasive procedures, which could be discomforting and carry risks for patients. In contrast, MRIs are a cost-effective and readily accessible modality in clinical settings.

**Fig 1:**
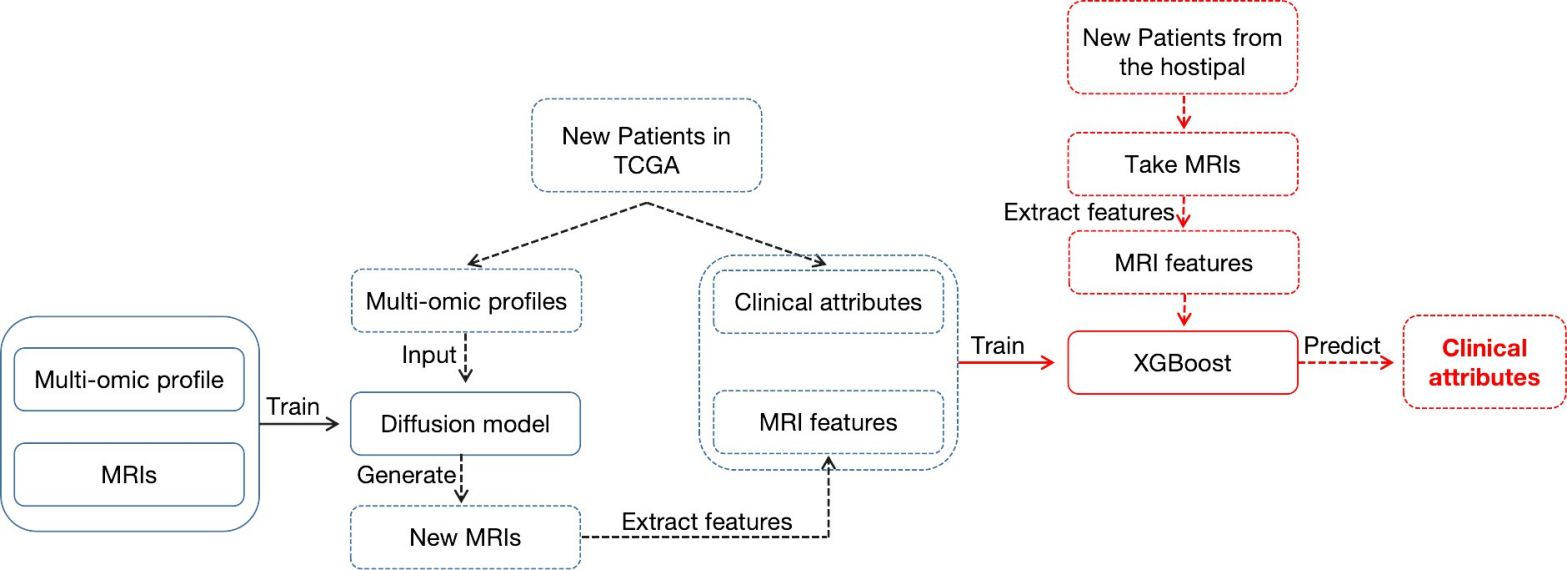
The overall workflow of the study.

Thus, in this study, our overarching goal is to leverage synthetic MRIs to acquire patients’ clinical attributes through radiogenomic studies. To be more specific, we first developed a powerful deep generative Conditional Probabilistic Diffusion Model (CPDM) to synthesize image data comparable to real patients’ MRIs. Subsequently, we used highly realistic synthetic MRIs to predict the clinical attributes of BC patients. The pipeline of the project is illustrated in **Fig 1**. The success of this study would not only enhance therapeutic effectiveness but also improve the overall clinical healthcare experience.

## 2. Material and Methodology

### 2.1. CPDM

**Fig 2:**
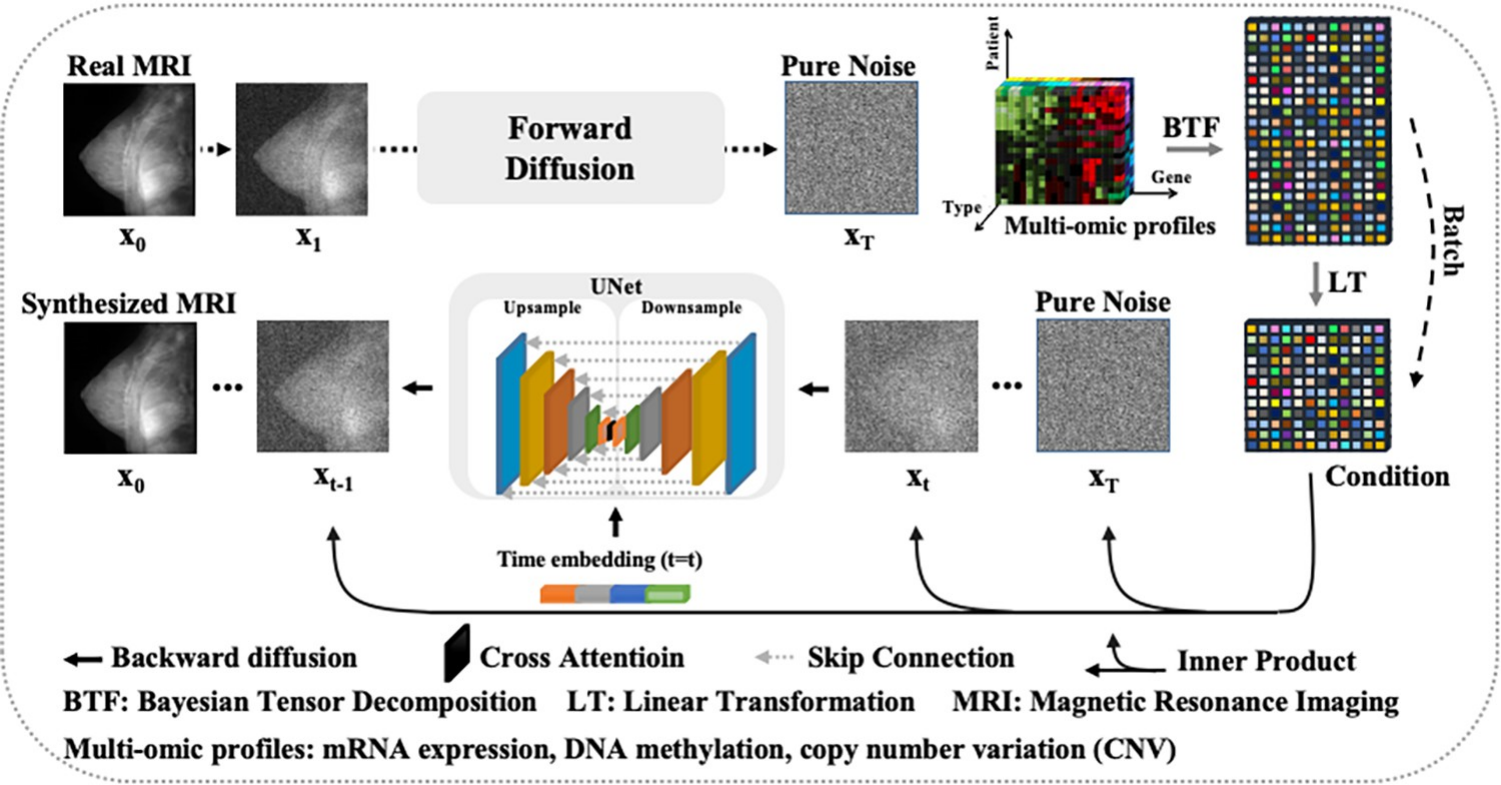
The architecture of the CPDM. The CPDM involves three core components, forward diffusion, condition preparation, and backward diffusion. In the forward diffusion, the model adds noise to the real MRI T times until a pure noise image is obtained. The collected multi-omic profiles are processed by BTF and produce a decomposed feature entity. Each row of the entity represents a multi-omic feature for a patient, which is used to guide the image generation in the backward diffusion. In the backward diffusion, the model computes an inner product of the image and the multi-omic feature (linearly extended to match the image shapes) and adds it back to the noised images. Then, the model consistently removes noise from the noised image, until getting the new synthesized MRIs. The noise is predicted by an UNet module with a cross-attention layer between upsampling and downsampling modules.

A CPDM was designed to generate MRIs from random noises, conditioned by a genomic profile. The implementation included two core steps. The first step was adding noises into an MRI until it was degraded to a pure noise image. The second step was reversing the first step under a genomic condition to denoise a pure noise image. These two processes were named forward diffusion and backward diffusion, respectively. **Fig 2** shows the architecture of the CPDM.

#### 2.1.1. Data collection and preprocessing

We collected the paired 58 sagittal MRIs and the corresponding multi-omic profiles (mRNA gene expression, DNA methylation, and copy number variation) of the patients from TCIA-BRCA and TCGA-BRCA projects. Since we would use a 15-fold cross-validation to train and test the models, each fold would have approximate 4 independent samples in the test set and 54 samples in the training set. The collected 3D sagittal MRIs comprised varying number of slices. To make it easy to interpret and perform visual inspections, these 3D MRIs were represented as 2D orthographic projections [25–26]. Then, to reduce computational complexity, these MRIs were resized to 128 × 128 by utilizing the nearest pixel method [27]. There were also some steps to process the multi-omic data. After collecting the raw multi-omic data, samples or features with all-zero values were removed to ensure data quality. Then, to focus on the most variable and informative features, the top 10 percent of the genes with the largest Coefficient of Variation (CV) were selected. This filtering process resulted in retaining 4515 genes for 754 patients in the final data matrices. The data matrices for each patient include a 2D mRNA gene expression matrix, a 2D DNA methylation matrix, and a 2D copy number variation matrix. These data matrices were then used to construct a 3D tensor, serving as the input for Bayesian tensor factorization (BTF) [28]. The inputted 3D tensor was finally decomposed into a 2D matrix with 17 latent factors for each patient. To further analyze the adaptive ability of the model, we also collected a set of gene expressions of 123 ER+/HER2+ BC patients from the TCIA-BRCA and the TCGA-BRCA projects to repeat the experiment.

#### 2.1.2. Forward diffusion

**Fig 3:**
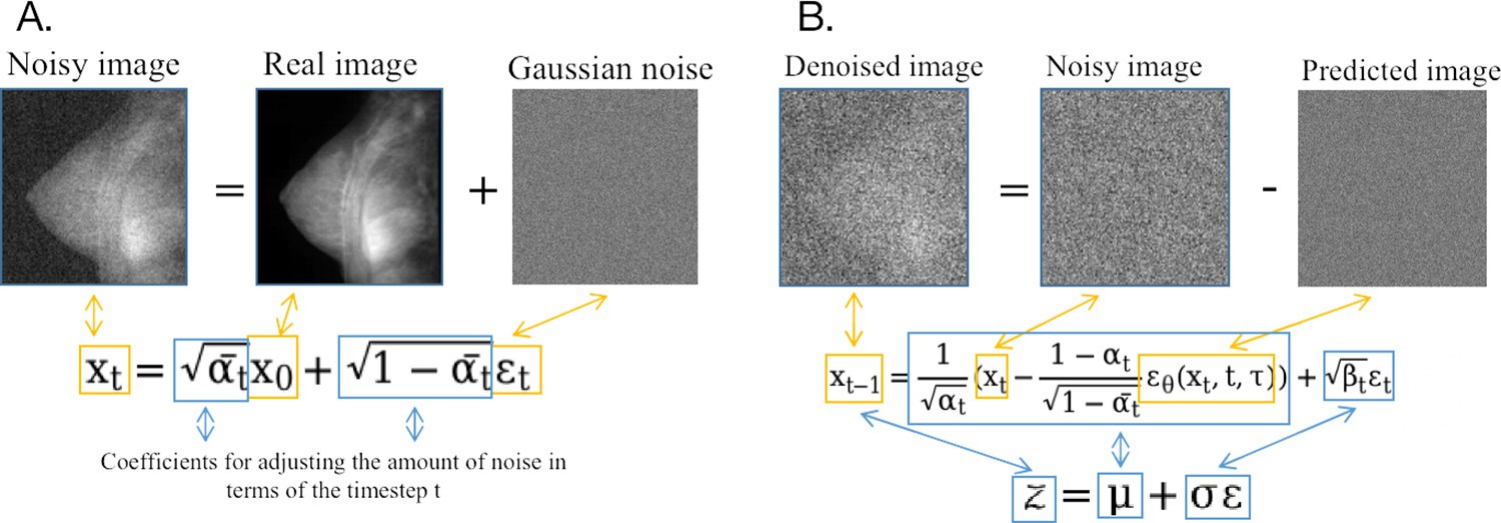
Diffusion procedure. **A.** Forward diffusion. **B.** Backward diffusion.

MRIs were degraded to pure noise images in the forward diffusion process. We presented the MRIs as x_0_ and pure noise images as x_T_, where T was the total steps assigned to degrade an MRI. The process of reasoning x_T_ was not instantaneous. Designing a Markov chain to obtain intermediate states x_t_ as transitions was the common strategy [16]. Specifically, due to the convenience of Gaussian noise in sampling data distributions, the model would add scheduled Gaussian noise into the sample to obtain a state at the deeper time step [29]. Mathematically, this could be represented as $q(x_{t}|x_{t-1})=N(x_{t};\sqrt{1-\beta_{t}}x_{t-1},\beta_{t}I)$, where x_t_ was the distribution of the current noised image, x_t-1_ was the distribution of the previous noised image, β_t_ was the variance schedule, 0<β_t_<1, β_0_ was the smallest number and β_T_ was the largest number and I was an identity matrix. Based on the definition of the Gaussian noise, q(x_t_|x_t−1_) could be further reparametrized as

$$x_{t}=\sqrt{1-\beta_{t}}x_{t-1}+\sqrt{\beta_{t}}\varepsilon_{t-1}$$ (1),

where ε∈N(0,1). Let $\alpha_{t}=1-\beta_{t},\bar{\alpha_{t}}=\prod_{i=1}^{t}\alpha_{i},\varepsilon_{1},\varepsilon_{2},\ldots ,\varepsilon_{t-1}∼N(0,I)$ and $\bar{\varepsilon_{1}},\bar{\varepsilon_{2}},\ldots ,{\bar{\varepsilon }}_{t-1}∼N(0,I)$. The **Eq 1.** could be substituted as

$$x_{t}=\sqrt{\alpha_{t}\alpha_{t-1}}x_{t-2}+\sqrt{\alpha_{t}{(1-\alpha }_{t-1})}\varepsilon_{t-2}+\sqrt{{1-\alpha }_{t}}\varepsilon_{t-1}$$ (2),

which was able to be further merged as

$$x_{t}=\sqrt{\alpha_{t}\alpha_{t-1}}x_{t-2}+\sqrt{{1-\alpha }_{t}\alpha_{t-1}}{\bar{\varepsilon }}_{t-2}$$

$$=\sqrt{\alpha_{t}\alpha_{t-1}\ldots \alpha_{1}}x_{0}+\sqrt{1-\alpha_{t}\alpha_{t-1}{\ldots \alpha }_{1}}\varepsilon_{t}$$

$$=\sqrt{{\bar{\alpha }}_{t}}x_{0}+\sqrt{1-{\bar{\alpha }}_{t}}\varepsilon_{t}$$ (3),

representing any intermediate states x_t_ in terms of the inputted MRI x_0_. **Fig 3A** illustrated this process.

#### 2.1.3. Backward diffusion

In the backward diffusion process, a new MRI would be synthesized from a pure Gaussian noise. In mathematical terms, the distribution of the synthesized MRI was represented as q(x_t−1_|x_t_, x_0_). Due to the irreversible nature of the Markov chains, the strategy using to solve q(x_t−1_|x_t_, x_0_) was to train a deep neural network model (U-Net based framework) to approximate the distribution of it. Before using U-Net framework to approximate the data distribution, employing Bayes’ rule to q(x_t−1_|x_t_, x_0_), it would give

$$q(x_{t-1}|x_{t},x_{0})=q(x_{t}|x_{t-1},x_{0})\frac{q(x_{t-1}|x_{0})}{q(x_{t}|x_{0})}$$

$$\propto \exp\left(-\frac{1}{2}(\frac{{\left(x_{t}-\sqrt{\alpha_{t}}x_{t-1}\right)}^{2}}{\beta_{t}}+\frac{{\left(x_{t-1}-\sqrt{{\bar{\alpha }}_{t-1}}x_{0}\right)}^{2}}{1-{\bar{\alpha }}_{t-1}}-\frac{{\left(x_{t}-\sqrt{{\bar{\alpha }}_{t}}x_{0}\right)}^{2}}{1-\bar{\alpha_{t}}})\right)$$

$$=exp\left(-\frac{1}{2}(\left(\frac{\alpha_{t}}{\beta_{t}}+\frac{1}{1-{\bar{\alpha }}_{t-1}}){x^{2}}_{t-1}-2(\frac{\sqrt{\alpha_{t}}}{\beta_{t}}x_{t}+\frac{\sqrt{{\bar{\alpha }}_{t-1}}}{1-{\bar{\alpha }}_{t-1}}x_{0})x_{t-1}+C(x_{t},x_{0}\right))\right)$$ (4),

where C(x_t_, x_0_) was a constant. Referred to the **Eq 4.**, the mean $\tilde{\mu_{t}}(x_{t},x_{0})$ and variance $\tilde{\beta_{t}}$ of q(x_t−1_|x_t_, x_0_) could be parameterized as

$$\tilde{\beta_{t}}=1/(\frac{\alpha_{t}}{\beta_{t}}+\frac{1}{1-{\bar{\alpha }}_{t-1}})=\frac{1-{\bar{\alpha }}_{t-1}}{1-\bar{\alpha_{t}}}\beta_{t}$$ (5)

and

$$\tilde{\mu_{t}}(x_{t},x_{0})=(\frac{\sqrt{\alpha_{t}}}{\beta_{t}}x_{t}+\frac{\sqrt{{\bar{\alpha }}_{t-1}}}{1-{\bar{\alpha }}_{t-1}}x_{0})/(\frac{\alpha_{t}}{\beta_{t}}+\frac{1}{1-{\bar{\alpha }}_{t-1}})$$

$$=\frac{\sqrt{\alpha_{t}}(1-{\bar{\alpha }}_{t-1})}{1-{\bar{\alpha }}_{t}}x_{t}+\frac{\sqrt{{\bar{\alpha }}_{t-1}}\beta_{t}}{1-{\bar{\alpha }}_{t}}x_{0}$$ (6)

according to the standard Gaussian density function. Reformatting the **Eq 3.**, x_0_ could be expressed as **Eq 7.**


$$x_{0}=\frac{1}{\sqrt{{\bar{\alpha }}_{t}}}(x_{t}-\sqrt{1-{\bar{\alpha }}_{t}}\varepsilon_{t})$$ (7)

Referring to **Eq 6.** and **Eq 7.**, $\tilde{\mu_{t}}$ could be further conceptualized as **Eq 8.**


$${\tilde{\mu }}_{t}(x_{t})=\frac{1}{\sqrt{\alpha_{t}}}(x_{t}-\frac{1-\alpha_{t}}{\sqrt{1-{\bar{\alpha }}_{t}}}\varepsilon_{t})$$ (8)

According to the **Eq 8.** the mean μ_θ_ of the neural network-approximated distribution q(x_t−1_|x_t_, x_0_) could be constructed as

$$\mu_{\theta }(x_{t},t,\tau )=\frac{1}{\sqrt{\alpha_{t}}}\left(x_{t}-\frac{1-\alpha_{t}}{\sqrt{1-{\bar{\alpha }}_{t}}}\varepsilon_{\theta }(x_{t},t,\tau )\right)$$ (9),

where ε_θ_ was the noise predicted by the deep neural network and τ was the condition. The loss function of the deep neural network would then be defined as **Eq 10. Fig 3B** illustrated the process of removing noise from noisy image in the backward diffusion.


$$L=E_{t∼[1,T],x_{0},\varepsilon_{t},\tau }[\frac{1}{2{\sigma_{t}}^{2}}∥{\tilde{\mu }}_{t}(x_{t})-\mu_{\theta }(x_{t},t,\tau ){∥}^{2}]$$

$$=E_{t∼[1,T],x_{0},\varepsilon_{t},\tau }\left[\frac{(1{-\alpha_{t})}^{2}}{2{\sigma_{t}}^{2}\alpha_{t}(1-{\bar{\alpha }}_{t})}∥\varepsilon_{t}-\varepsilon_{\theta }(x_{t},t,\tau ){∥}^{2}\right]$$

$$=E_{t∼[1,T],x_{0},\varepsilon_{t},\tau }[∥\varepsilon_{t}-\varepsilon_{\theta }(\sqrt{{\bar{\alpha }}_{t}}x_{0}+\sqrt{1-{\bar{\alpha }}_{t}}\varepsilon_{t},t,\tau ){∥}^{2}]$$ (10)

**Table 1** Algorithm 1 showed the pseudo-code of the training the CPDM and **Table 1** Algorithm 2 showed the pseudo-code of synthesizing new MRIs according to the genomic information of patients.

**Table 1:** Pseudo-codes for the CPDM.

	**Algorithm 1**
1	**repeat**
2	x_0_ ~ q(x_0_)
3	t ~ Uniform({1,. . .,T})
4	ε∼N(0,I)
5	Take gradient descent step on $\nabla_{\theta }[∥\varepsilon_{t}-\varepsilon_{\theta }(\sqrt{{\bar{\alpha }}_{t}}x_{0}+\sqrt{1-{\bar{\alpha }}_{t}}\varepsilon_{t},t,\tau ){∥}^{2}]$
6	**until** converged
	**Algorithm 2**
1	$x_{T}∼N(0,I)$, t = T
2	while t≥1 do
3	z∼N(0,I)ift>1,elsez=0
4	$x_{t-1}=\frac{1}{\sqrt{\alpha_{t}}}(x_{t}-\frac{1-\alpha_{t}}{\sqrt{1-{\bar{\alpha }}_{t}}}\varepsilon_{\theta }(x_{t},t,\tau ))+\sigma_{t}z$
5	t = t-1
6	return x_0_

*Recap some key notations: x_0_: inputted MRIs, x_T_: pure noise images, x_t_: intermediate states, T: timesteps, t: a middle timestep, ε_θ_: the deep neural network and τ: condition (genomic information).

#### 2.1.4. Conditioning

*2*.*1*.*4*.*1*. *Cross-attention*. We strategically placed the cross-attention module between the downsampling and upsampling processes of the U-Net model to align the imaging information with the multi-omic information [30]. The cross-attention mechanism allowed the model to attend to relevant parts of the MRIs (say, modality B) based on the content of the multi-omics data (say, modality A), enabling the generation of MRI projections that match the multi-omic data. The essence of the cross-attention mechanism was interacting the query vectors from modality A with the key vectors from modality B to compute attention scores. These attention scores were then used to weight the value vectors from modality B, to capture the cross-modal interactions between these two modalities. Mathematically, the query vector for the modality A could be represented as $Q_{A}=X_{A}∙W_{Q}^{A}$, where *X*_*A*_ is the representation of the modality A and $W_{Q}^{A}$ is a learnable weight matrix. Similarly, the key vector for modality B could be represented as $K_{B}=X_{B}∙W_{K}^{B}$, and the value vector for modality B could be represented as $V_{B}=X_{B}∙W_{V}^{B}$, where *X*_*B*_ is the representation of the modality B, and $W_{K}^{B}$ and $W_{V}^{B}$ are learnable weight matrices. Then, the attention scores (A) from modality A to modality B could be calculated by

$$A=softmax\left(\frac{Q_{A}∙K_{B}^{T}}{\sqrt{d_{k}}}\right)$$ (11),

where $\sqrt{d_{k}}$ is the dimension of the key vectors. The attention scores of modality A were used to calculate the weighted aggregation of the value vectors (WV) of modality B. The weighted value vectors could then be calculated as the dot product of the attention scores of modality A and the value vector for modality B, i.e. *WV* = *A*∙*V*_*B*_. In the end, we computed the final output obtained from the cross-attention mechanism after aggregating the information from modality B using the attention scores computed from modality A, which could be used as input for subsequent layers. We call this a fused representation (FR) and

$$FR=WV∙W_{O}^{B}$$ (12),

where $W_{O}^{B}$ is an additional learnable weight matrix for the aggregation.

*2*.*1*.*4*.*2*. *Integration of multi-omic and MRI data*. To facilitate the synthesis of MRI projections that were both visually faithful and biologically significant, we used the inner product to integrate the compact multi-omic feature vectors, denoted as F, with detailed MRI projection images, denoted as P. In mathematics, the inner product X involved calculating the dot product between the genomic information vector and the MRI projection image [31]. So, we had

$$X_{ij}=F∙P=\sum_{k=1}^{n}F_{ik}{\cdot P}_{kj}$$ (13),

where *X*_*ij*_ represents each element in the result of the inner product, F and P were feature matrix and image matrix both with size *n*×*n*, *F*_*ik*_ and *P*_*kj*_ represented the individual elements of the feature vector and image, respectively. This operation resulted in a matrix that encapsulates the correlations and interactions between the two data modalities. Each value in the resulting matrix represented the cumulative contribution of the corresponding elements from the multi-omics feature vector and the image. This matrix encoded the alignment and correlation between the genomic features and the MRIs. This matrix could then be used as a fusion mechanism to guide the synthesis of MRIs. We implemented the fusion mechanism by adding our computed inner product into the image matrix to update the Noisy Input (NI) of the model, i.e.

NI=P+X (14),

where P was the noisy image and X was the inner product of the multi-omic feature vector and the image vector. This encapsulated both the molecular insights from the feature vectors and the visual information from the images [32]. Finally, both the noisy input and the feature vector were treated as the input of the CPDM.

#### 2.1.5. CPDM evaluation

The evaluation of CPDM was based on three performance metrics: Fréchet inception distance (FID), Mean Square Error (MSE) and Structural Similarity Index Measure (SSIM). FID was used to compare the similarity between the real image and the synthetic image by calculating the Fréchet distance. FID was articulated as

$$FID(x,g)={\left\|\mu_{x}-\mu_{g}\right\|}^{2}+Tr\left(\sum_{x}+\sum_{g}-2{\left(\sum_{x}\sum_{g}\right)}^{\frac{1}{2}}\right)$$ (15),

where *μ*_*x*_, *μ*_*g*_ represented the feature-wise mean of the real and synthetic images, while ∑_*x*_, ∑_*g*_ were their covariance matrices [33]. A low FID score indicated a high degree of similarity between images. Achieving a score of 0.00 signifies that the two images were identical. We also calculated the Fréchet Inception Distance STandard Deviation (FID-STD) to evaluate the consistency and stability of the CPDM. This metric could assess the variation in the model’s performance across different runs or image batches. A lower FID-STD value indicated a higher degree of consistency in the quality of images generated by the CPDM, signifying its reliability. MSE quantified the average squared difference between estimated values and actual values and was extensively utilized in image reconstruction tasks. MSE emphasizes the accuracy of the reconstructed images, which was given by

$$MSE(Ι,\hat{Ι})=\frac{1}{n}\sum_{i=1}^{n}{\left({Ι}_{i}-{\hat{Ι}}_{i}\right)}^{2}$$ (16),

where *I*_*i*_, ${\hat{I}}_{i}$ were the pixel values of the real and generated images, respectively, and n was the number of pixels [34]. A lower MSE value was indicative of superior performance. SSIM was a metric designed to assess the perceived quality of images and their similarity. Differing from FID, SSIM specifically evaluates changes in luminance, contrast, and structure between two images, providing a score that ranges from -1 to 1. SSIM was defined as

$$SSIM(I,\hat{I})=\frac{\left(2\mu_{I}\mu_{\hat{I}}+c_{1})(2\sigma_{I\hat{I}}+c_{2}\right)}{\left({\mu_{I}}^{2}+{\mu_{\hat{I}}}^{2}+c_{1})({\sigma_{I}}^{2}+{\sigma_{\hat{I}}}^{2}+c_{2}\right)}$$ (17),

where μ_I_, $\mu_{\hat{I}}$ were the average intensities, σ_I_^2^,${\sigma_{\hat{I}}}^{2}$ were the variances, $\sigma_{I\hat{I}}$ was the covariance, and c_1_, c_2_ were constants [35]. A higher SSIM value indicates not only greater similarity between images but also an enhanced perceived quality.

### 2.2. Applications using synthetic radiomic data

#### 2.2.1. Data collection and preprocessing

The data collection process involved aggregating mutation status, ER status, survival, and ER+/HER2+ data from the TCGA database (https://www.cbioportal.org/). We prepared datasets for clinical analysis by matching labels to the synthesized MRIs based on the available paired clinical data. **Table 2** presents the details of the datasets.

**Table 2:** The data distribution and details of datasets used to train XGBoost models.

Item	Total	Negatives	Positives
TP53 mutation status (multi-omic)	754	502	252
ER status (multi-omic)	708	164	544
ER+/HER2+ (multi-omic)	66	26	40
ER+/HER2+ (gene expression)	123	60	63
Survival for all patients (multi-omic)	740	-	-
Survival for ER+/HER2+ patients (multi-omic)	66	-	-
Survival for ER+/HER2+ patients (gene expression)	123	-	-

There are 754 patients with both multi-omic profiles and TP53 mutation status. Among them, 252 patients exhibited mutations in the TP53 gene, designated as 1, while the remaining 502 patients with normal genetic profiles were labeled as 0. For ER status, there are 708 patients with both ER status and multi-omics profiles. Among them, 544 are positive while 164 are negative. For the 66 ER+/HER2+ BCs with multi-omics profiles, 26 of them are from the Subgroup 1 and the other 40 are from the Subgroup 2. Among the 123 ER+/HER2+ BCs with gene expression data, 63 of them belong to the Subgroup 1 while the other 60 belong to the Subgroup 2. Subgroup 1 and subgroup 2 are the further categorizations for ER+/HER2+ BC [36]. The patients from these two subgroups usually have different treatment responses and clinical outcomes.

The approach for the survival dataset entailed compiling patients with recorded survival data, encompassing survival days and outcomes (survival or deceased) and matched MRIs. Considering all 754 patients who had generated MRIs, there were 740 patients with survival data. For the 123 patients who had ER+/HER2+ Subtype data, we could obtain 66 patients who had multi-omic profile-based MRIs and 123 patients with gene expression-based MRIs. This process ensured that the analyses were grounded in robust data intersections, offering a robust foundation for the subsequent analyses.

#### 2.2.2. Classification and prediction

For binary classification tasks, namely the prediction of mutation status, ER status and ER+/HER2+ subtypes, our approach entailed extracting image features using well-established tools such as PyRadiomics and pre-trained CNN models, including VGG16, ResNet50, and InceptionV3 [37–40]. Due to the complexity of the radiomics BC study, we identified the most proper extracting method in these commonly used tools via experiments. **S1 Table** shows the number of features extracted from MRIs generated according to the multi-omic profiles and gene expressions, respectively. These features were then employed as input to train XGBoost models, optimized through the RandomizedSearchCV tool [41–42].

Innovatively, we extended the utility of the extracted image features for survival analysis through the adoption of tools such as DeepSurv and CoxPHFilter [43–44]. By utilizing this strategy, we explored new dimensions of patient prognosis.

#### 2.2.3. Evaluations

In the classification task, the model evaluation results were based on the 10-fold cross-validation method. This process involved partitioning the training set into 10 equal batches. In each validation cycle, one batch was designated as the test set, and the remaining segments were amalgamated to form the training set. The model was trained and tested sequentially across all 10 folds. The overall performance of the model was then ascertained by averaging the outcomes from all 10 tests, ensuring a comprehensive assessment that leverages every data point for both training and validation.

Receiver operating characteristic (ROC) curves, area under the receiver operating characteristic curve (AUROC), precision-recall curves, area under the precision-recall curve (AUPRC), and the F1 score were used to evaluate the performance of XGBoost models. The ROC curve was a graphical representation of a classifier’s performance, plotting the true positive rate against the false positive rate across various decision thresholds. The AUROC (the full mark was 1.00) quantified the area under the ROC curve, with a higher value indicating better classification performance. On the other hand, the precision-recall curve plotted precision against the recall, focusing on the trade-off between accurately identified positive cases (precision) and the total actual positive cases captured (recall). The AUPRC (the full mark was 1.00) reflected the area under this curve, providing a measure of a model’s ability to balance precision and recall. The F1 score was the harmonic mean of precision and recall. The F1 score combined both precision and recall into a single metric, making it useful for evaluating models in scenarios where the class imbalance is present. A higher F1 score (the full mark was 1.00) indicates a better balance between precision and recall.

For the survival analysis tasks, the Concordance-index (C-index) and the p-value from the log-rank test were used to evaluate the performance of the survival models. The C-index stood as a fundamental metric widely employed in survival analysis and medical investigations to assess the effectiveness of predictive models in the context of time-to-event outcomes [45–46]. It served as a yardstick for measuring a model’s competence in correctly ordering pairs of observations with and without events according to their actual survival times. Spanning from 0.5 to 1.0, the C-index offered a concise yet informative gauge of a model’s capability to discriminate between patients exhibiting varying survival durations. By capturing the nuanced interplay between predictors and survival outcomes, the C-index assumed a pivotal role in appraising the predictive performance of models in the realm of medical research. Higher c-index values denoted stronger predictive abilities, indicating a model’s success in accurately ranking patients based on their actual survival times. The p-value from the log-rank test evaluates the other aspect of the survival models, such as the shape of the survival curves, providing information that the C-index may not capture [47]. The log-rank test p-value tests the null hypothesis that there is no difference between the survival curves of different groups. A lower log-rank test p-value (less than or equal to 0.05) indicates a statistically significant difference in survival distributions, thereby demonstrating the models’ ability to distinguish between patient groups with different survival patterns.

## 3. Results

### 3.1. Results of CPDMs

#### 3.1.1. Data collection and preprocessing

In each fold, the 54 samples from the training set consisting of paired multi-omic profiles and real MRI projections were used to train the CPDM, and 4 samples also with paired multi-omic profiles and real MRI projections in the test sets were used to test the performance of the trained model. Then, there were 726 patients with solely multi-omic profiles that could be collected from the TCIA-BRCA project, which could be used to guide MRI projection synthesis.

#### 3.1.2. Model training

To train a CPDM so it could generate MRI projections according to patients’ multi-omic profiles, we performed iteration steps on the training set consisting of paired MRI projections and multi-omic profiles until the loss was converged. **S1A and S1B Fig** showed the variation trend of the loss values of the model with the epoch increases. From both figures, along with the increment of the epoch, the losses of the models were decreasing and finally fluctuate between small intervals, reaching convergence. Based on the diagrams, it could be concluded that models have small and stable loss values after 1100 rounds of iteration, which could approximate the real sample distribution well during the denoising process. **S2 and S3 Tables** (the bold items were the finalized configurations) respectively showed 7 hyperparameters for CPDM optimization for the multi-omic profile version and gene expression version.

In addition, we also trained four classic deep generative models AEs, VAEs, Transformers, and GANs, using the same datasets. These comparisons were conducted to benchmark our model against these established standards, employing consistent performance metrics to critically evaluate and understand the unique strengths and limitations of each approach, thereby highlighting the advancements our models bring to the field.

#### 3.1.3. Model performance

**Table 3** showed the training and testing performances of four baseline models and our CPDMs on the multi-omic dataset and the gene expression dataset. Each model was evaluated based on the four performance metrics. The calculations for FID, FID-STD, MSE, and SSIM were conducted following standardized protocols. From the table, three classical generative models trained on different datasets in this task all yielded results with lower performances than CPDM, especially low SSIM scores (all below 0.5). This reflects the advanced performance of CPDM compared to traditional generative models.

**Table 3:** Performance metrics.

		FID		STD-FID		MSE (×10^−2^)		SSIM	
Model	Dataset	Train	Test	Train	Test	Train	Test	Train	Test
AE	multi-omic	5.64	5.76	1.67	2.05	5.42	5.52	0.17	0.16
	gene expression	5.66	5.75	1.67	1.99	5.43	5.50	0.18	0.16
VAE	multi-omic	3.91	5.22	0.66	4.04	2.80	4.51	0.18	0.19
	gene expression	2.19	3.63	**0.19**	1.84	2.13	3.31	0.17	0.18
Transformer	multi-omic	4.19	4.27	0.75	1.11	4.46	4.57	0.32	0.28
	gene expression	4.66	4.82	1.24	1.35	4.84	5.05	0.26	0.21
GAN	multi-omic	15.24	16.19	3.83	4.16	14.28	15.69	0.08	0.07
	gene expression	12.72	13.40	3.10	3.60	11.02	11.63	0.09	0.09
CPDM (Ours)	multi-omic	3.59	3.11	0.48	**0.74**	3.63	3.15	0.58	**0.59**
	gene expression	**1.26**	**2.02**	0.64	0.86	**1.31**	**2.08**	**0.64**	**0.59**

*Note: The bold numbers in the table indicate the best performance under each metric among the different models.

**Fig 4:**
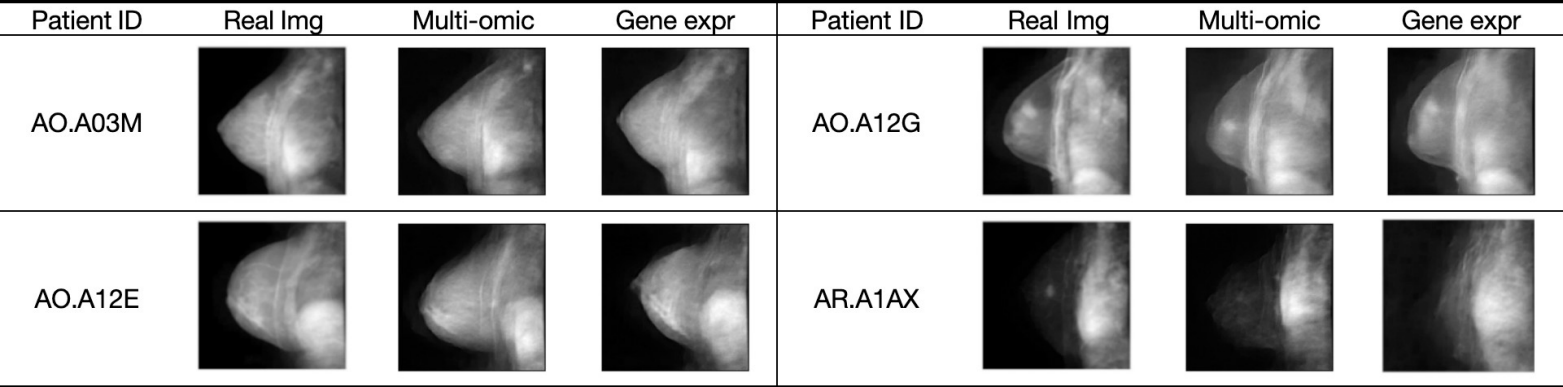
The results on the test set. The figure presented results for four patients included in the test set. Each patient was shown with three images: the real clinical image (Real Img), the image synthesized from their multi-omic profiles (Multi-omic), and the image synthesized from their gene expressions (Gene expr).

To further evaluate the quality of the synthetic images and the performance of the CPDM, we conducted a visual inspection of synthetic images on the test sets. **Fig 4** demonstrated the visualized results of the multi-omic and gene expression versions, respectively. In each table, for each patient in the test set, the corresponding real MRI images from the database were displayed alongside a CPDM-synthesized MRI image. Displayed images allowed the comparison of the similarity between real and synthesized images. These comparisons revealed that the MRIs synthesized by the multi-omic data-based CPDM and gene expression data-based CPDM closely aligned with the real MRIs in content and quality, further corroborating the exceptional performance of the CPDMs.

**Fig 5:**
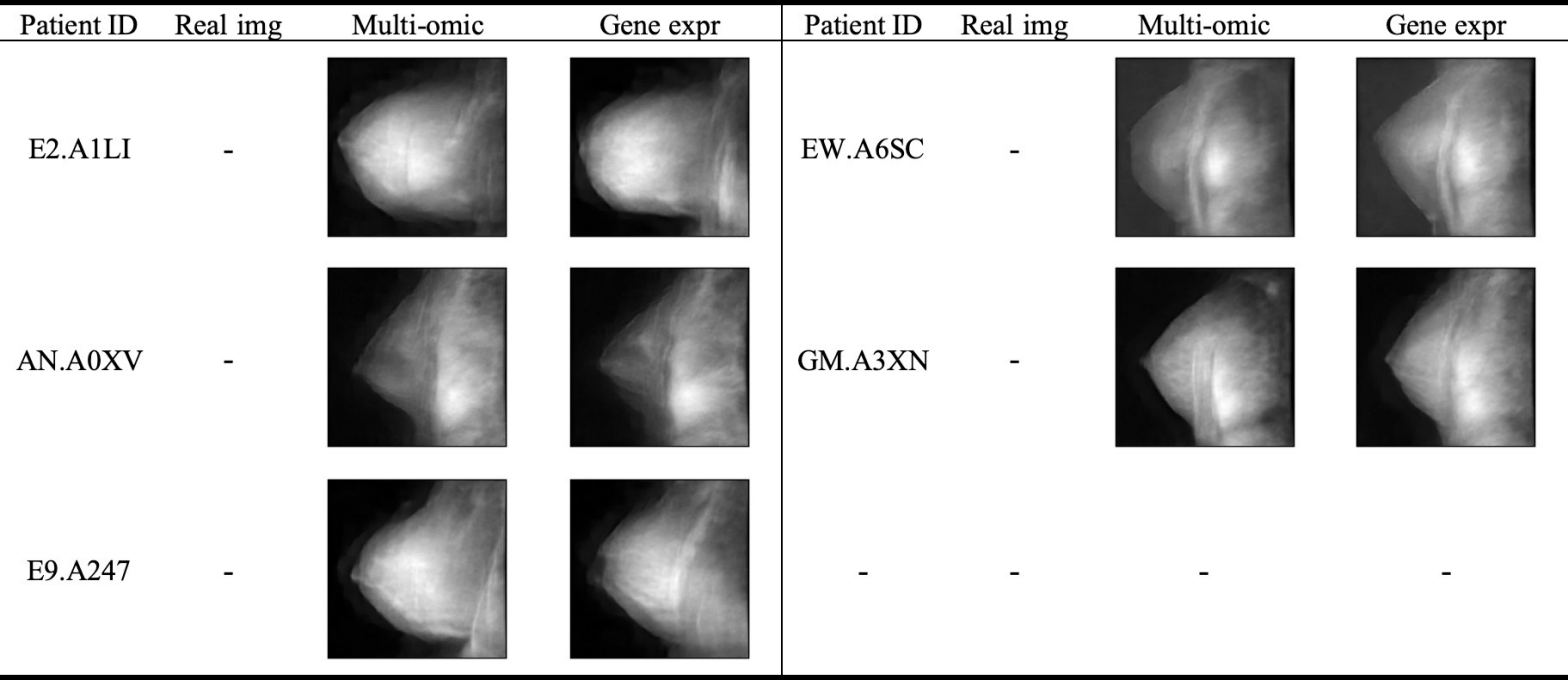
Synthetic images from unpaired data. The figure showed synthetic images for five patients who had genomic data but no real clinical images. The three patients on the left were selected by clustering their multi-omic profiles, while the two on the right were selected by clustering their gene expressions. For each patient, an image synthesized from their multi-omic profiles and an image synthesized from their gene expressions were presented.

Due to the lack of real BC MRI images for patients in the unpaired dataset, we could not employ numerical evaluation for synthetic images. Instead, we resorted to visual inspection, which included inspecting the visual quality of the synthesized images, as well as the similarity between MRIs synthesized by multi-omic profile-based CPDM and gene expression-based CPDM. **Fig 5** presented the synthetic results for 5 patients. The patients were selected according to the K-means clustering results of their multi-omic profiles and gene expressions, as shown in **S2A and S2B Fig** [48]. Through the visual inspections, it could be observed that MRIs synthesized based on the unpaired dataset exhibited granularity as close as real MRIs in the paired dataset. The synthesized MRIs always displayed distinct breast contours and clear tissue structures and could always bring out rich varieties based on the different input features. Moreover, MRIs for an identical patient synthesized by multi-omic data-based CPDM and gene expression data-based CPDM showed similarity in overall structure and detail. Additionally, we also used computational tools, including CharCPT-4 and x-ray interpreter, to further analyze the synthesized images and utilized the patients’ clinical data from TCGA-BRCA to validate the analysis results [49–50]. **S4 and S5 Tables** presented the results of using the computational tools to analyze the synthetic images. The findings from the computational tools for the synthetic images were consistent with the actual clinical data of the patients, suggesting the reliability of these images. The performance reflects the success of the CPDM in this task, especially its robust generalization capability.

### 3.2. Results of applications

#### 3.2.1. Data collection and preprocessing

The well-trained CPDM was then used to generate 754 (726 patients with unpaired data + 28 patients with paired data) MRIs according to patients’ multi-omic profiles. This step was repeated on the 123 HER2+/ER+ BCs’ gene expression dataset and got 123 synthetic MRIs. These synthetic images were used to predict the mutation status of BC driver genes, BC ER status, BC ER+/HER2+ subtypes, and survival information.

#### 3.2.2. Models training

**S6 and S7 Tables** showed the hyper-parameter tuning results of the XGBoost model for the TP53 mutation status prediction task using the MRI features extracted by the ResNet50 model and ER status prediction task using the MRI features extracted by the PyRadiomics tool, respectively. **S8 Table** showed the hyper-parameter tuning results of the XGBoost model for the ER+/HER2+ subtype prediction task using the MRI features extracted by the PyRadiomics tool.

**S9 Table** showed the hyper-parameter tuning results of the CoxPHfilter model for 740 patients with multi-omic-guided synthetic MRIs. The MRI features inputted into the model were extracted by the ResNet50 model. **S10 Table** showed the hyper-parameter tuning results of the CoxPHfilter model for 66 patients with ER+/HER2+ data. The data used to train this model was the features of multi-omic-guided synthetic MRIs, extracted by the ResNet50 model of these patients.

#### 3.2.3. Model performances

**Fig 6:**
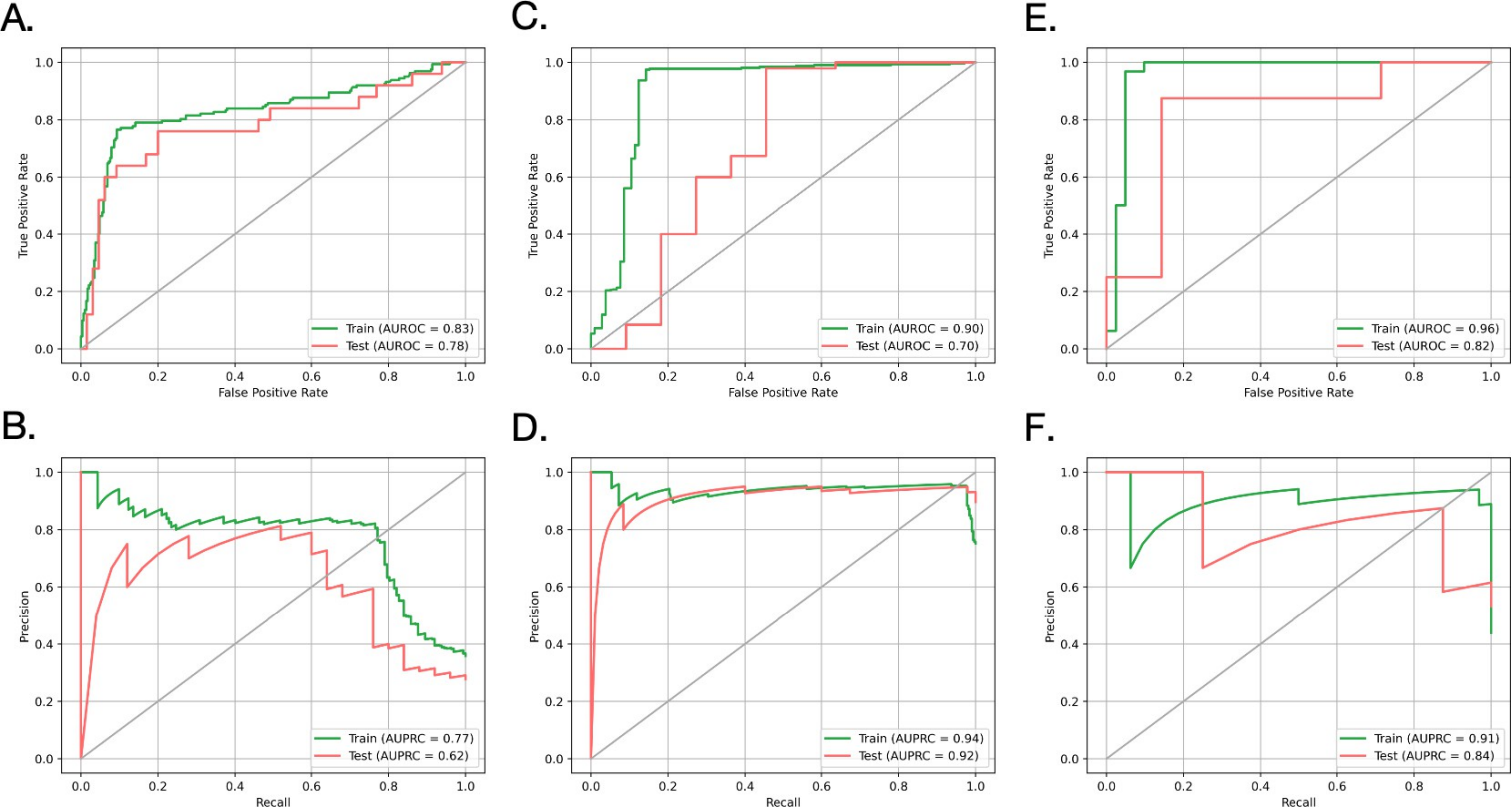
Average ROC and precision-recall curve for classification tasks from cross-validation. **A.** Average ROC curves of the gene TP53 mutation status prediction based on the XGBoost model trained on the MRI (multi-omic version) features extracted by the PyRadiomics tool. **B.** Average precision-recall curves of the gene TP53 mutation status prediction based on the XGBoost model trained on the MRI (multi-omic version) features extracted by the PyRadiomics tool. **C.** Average ROC curves of the ER status prediction based on the XGBoost model trained on the MRI (multi-omic version) features extracted by the PyRadiomics tool. **D.** Average precision-recall curves of the ER status prediction based on the XGBoost model trained on the MRI (multi-omic version) features extracted by the PyRadiomics tool. **E.** Average ROC curves of the ER+/HER2+ subtypes prediction based on the XGBoost model trained on the MRI (gene expresssion version) features extracted by the PyRadiomics tool. **F.** Average precision-recall curves of the ER+/HER2+ subtypes prediction based on the XGBoost model trained on the MRI (gene expresssion version) features extracted by the PyRadiomics tool.

*3*.*2*.*3*.*1*. *XGBoost model for TP53 mutation status and ER status prediction*. The TP53 mutation status module in **Table 4** showed the results of using different methods to predict the mutation status of the TP53 gene. Comparing these results with the baseline performance of TP53 mutation status prediction in **S11 Table**, it was observed that the synthetic MRI-based classification outcomes closely matched those derived from patients’ actual multi-omic profiles. This indicated that the synthetic MRI data encapsulated information that was almost consistent with the patients’ real multi-omic profiles. Furthermore, the AUPRCs for TP53 mutation status prediction in **Table 4** significantly exceeded the baseline AUPRCs shown in **S12 Table**, suggesting that the model had learned to recognize the positive cases and made reliable predictions in practice. Additionally, **Fig 6A** and **6B** illustrated the average ROC and precision-recall curves from cross-validation, respectively, further corroborating the classification capability of the model. These findings demonstrated the feasibility of predicting TP53 gene mutation status using synthetic MRIs.

**Table 4:** The results of the TP53 mutation status and ER status prediction.

		AUROC		AUPRC		F1 score	
		Train	Test	Train	Test	Train	Test
TP53 mutation status	PyRadiomics	0.83	**0.78**	**0.77**	**0.62**	**0.79**	0.67
	VGG16	**0.84**	0.72	0.73	0.56	0.77	**0.69**
	ResNet50	0.83	0.74	0.74	0.55	0.76	0.64
	InceptionV3	0.81	0.71	0.69	0.48	0.75	0.52
ER status	PyRadiomics	**0.90**	**0.70**	**0.94**	**0.92**	**0.96**	**0.94**
	VGG16	0.88	0.68	0.92	0.85	0.95	0.91
	ResNet50	**0.90**	0.62	0.92	0.88	0.95	0.90
	InceptionV3	**0.90**	0.62	**0.94**	0.85	0.95	0.88

*Note: The bold numbers in the table indicate the best performance under each metric among the different models.

The ER status module in **Table 4** presented the results of using different methods to predict the ER status of patients. The values in the table demonstrated that the AUPRC and F1 scores were comparable with the baseline results in **S11 Table** and the AUPRC notably exceeded the baseline AUROC in **S12 Table.** However, the test AUROC was substantially lower than the baselines, and there was also a nonnegligible drop in AUROC from the training set to the test set when using the synthetic images for classification. These discrepancies may be attributed to the unbalanced dataset and the potential inaccuracies of images synthesized by CPDM in some aspects caused by limited training images. **Fig 6C** and **6D** depicted the average ROC and precision-recall curves from cross-validation for the model trained on the MRI features extracted by the PyRadiomics tool, providing additional context for the numerical results. Overall, these findings indicated that the model already had a certain classification ability, but it still had some limitations compared with using real multi-omics to make predictions.

*3*.*2*.*3*.*2*. *XGBoost model for ER+/HER2+ subtype prediction*. **Table 5** showed the performance of different versions of the XGBoost model in predicting ER+/HER2+ subtypes. **Fig 6E** and **6F.** illustrated the average ROC and precision-recall curves from cross-validation. These performance metrics indicated the model performed well in both discrimination and accuracy. Specifically, a high AUROC score suggested the model effectively differentiates ER+/HER2+ subtypes, while a high AUPRC score indicated the model could identify positive cases well. A good F1 score revealed that the model established a well-balanced trade-off between precision and recall. Moreover, by comparing the AUPRCs with the baseline AUPRCs in **S12 Table**, it could be observed that the obtained AUPRCs were markedly better than baseline AUPRCs. Likewise, the performance in **Table 5** was competitive when compared to the baseline performance in **S11 Table.** Notably, the synthetic MRI-based classification results were superior to the baseline results based on patients’ actual multi-omic profiles presented in **S11 Table.** This improvement may be attributed to the richer information contained in images, enhancing the classification tasks. These observations collectively demonstrated the robustness of the model in predicting ER+/HER2+ subtypes. Additionally, we also plotted the SHapley Additive exPlanations (SHAP) diagrams in **S3 Fig** for the XGBoost models trained on MRI features extracted by the PyRadiomics tool [51]. From the diagram we could identify the features that were important for the subgroup prediction and enhance the interpretability of the model.

**Table 5:** The results of the ER+/HER2+ subgroup prediction task.

		AUROC		AUPRC		F1 score	
		Train	Test	Train	Test	Train	Test
Multi-omic version	PyRadiomics	**0.98**	**0.81**	**0.96**	**0.83**	**0.97**	0.60
	VGG16	0.94	0.71	0.89	0.75	0.90	0.57
	ResNet50	0.91	0.77	0.87	0.67	0.90	**0.73**
	InceptionV3	0.95	0.79	0.90	0.73	0.90	0.57
Gene expression version	PyRadiomics	**0.96**	**0.82**	**0.91**	**0.84**	**0.94**	**0.88**
	VGG16	0.94	0.73	0.84	0.79	**0.94**	0.63
	ResNet50	0.87	0.69	0.84	0.73	0.87	0.71
	InceptionV3	0.91	0.76	0.88	0.80	0.91	0.70

*Note: The bold numbers in the table indicate the best performance under each metric among the different models.

*3*.*2*.*3*.*3*. *Survival analysis*. **Table 6** presented the performance (C-index score and log-rank test p-value) of the DeepSurv model and the CoxPHfilter models trained on the multi-omic profile-guided synthetic MRI features. **Table 7** provided the performance of DeepSurv models and CoxPHfilter models trained for patients with ER+/HER2+ subtype data. The assessed C-index scores and log-rank test p-values from survival models on both training and test sets in the two tables, especially the models trained on MRI features extracted by ResNet50, demonstrated models’ potent capability in predicting patient prognosis. This could be further supported by their close alignment with the baseline performance shown in the **S13 Table.** Additionally, in **Tables 6** and **7**, compared to the CoxPHfilter model, DeepSurv showed slightly inferior performance. This may be attributed to the small dataset sizes available for deep learning in this task, hindering the ability of the model to effectively learn and generalize. What’s more, comparing the performance of the CoxPHfilter models on multi-omic and gene expression versions, it was observed that the performance in the multi-omic version was better than in the gene expression version. Nevertheless, the performance metrics of multi-omic data-based CPDM were lower than the CPDM based on gene expression. This indicated the image features influencing FID and SSIM scores may be independent from those used for predicting survival. This observation revealed the complex relationship between image performance metrics and the predictive capacity in survival analysis, suggesting differing roles of various features in survival prediction. Lastly, **S4 Fig** presented Kaplan-Meier plots to show the shapes of the survival curves, which the p-values of log-rank test may not fully illustrate.

**Table 6:** Performance of survival analysis for all patients with multi-omic profiles.

		C-index		Log-rank test p-value	
		Train	Test	Train	Test
PyRadiomics	DeepSurv	0.57	0.52	0.13	0.23
	CoxPHfilter	**0.62**	0.57	0.03	0.06
VGG16	DeepSurv	0.58	0.54	0.12	0.19
	CoxPHfilter	0.59	**0.58**	0.15	0.16
ResNet50	DeepSurv	0.58	0.53	0.07	0.17
	CoxPHfilter	0.60	**0.58**	**0.01**	**0.01**
InceptionV3	DeepSurv	0.55	0.54	0.11	0.18
	CoxPHfilter	0.60	**0.58**	0.06	0.07

*Note: The bold numbers in the table indicate the best performance under each metric among the different models.

**Table 7:** Performance of survival analysis for patients with ER+/HER2+ subtype data.

			C-index		Log-rank test p-value	
			Train	Test	Train	Test
Multi-omic version	PyRadiomics	DeepSurv	0.76	0.70	0.14	0.22
		CoxPHfilter	0.89	0.77	0.04	0.10
	VGG16	DeepSurv	0.58	0.53	0.32	0.37
		CoxPHfilter	0.68	0.61	0.05	0.13
	ResNet50	DeepSurv	0.69	0.63	0.13	0.24
		CoxPHfilter	**0.97**	**0.88**	**0.01**	**0.05**
	InceptionV3	DeepSurv	0.77	0.71	0.11	0.41
		CoxPHfilter	0.79	0.74	0.08	0.33
Gene expression version	PyRadiomics	DeepSurv	0.58	0.55	0.62	0.72
		CoxPHfilter	0.64	0.63	0.40	0.49
	VGG16	DeepSurv	0.58	0.56	0.34	0.40
		CoxPHfilter	**0.69**	0.64	0.16	0.20
	ResNet50	DeepSurv	0.64	0.63	0.17	0.22
		CoxPHfilter	**0.69**	**0.66**	**0.05**	**0.04**
	InceptionV3	DeepSurv	0.57	0.55	0.43	0.47
		CoxPHfilter	**0.69**	**0.66**	0.26	0.34

*Note: The bold numbers in the table indicate the best performance under each metric among the different models.

## 4. Discussion

### 4.1. Trained models on small datasets

The major challenge in this study was to train generative models on a small dataset. It was easy to cause the potential epistemic uncertainty and noise in the training process when training a generative model on a small dataset. Compared to the classic generative models, the success of CPDM trained on a small dataset could be attributed to the unique advantages of its probabilistic framework and the utilization of methods that simplified sample features.

The unique probabilistic framework of the CPDM allowed it to simulate the inherent uncertainty of data sample generation in the diffusion process. Specifically, when iterating the outliers in the data, they would be treated as high uncertainty instances and assigned low probability densities, which reduced the interference of the model prediction results. Conversely, if the data point conformed to the overall distribution of training samples, it would obtain a high probability density and enhance the confidence of the prediction. Moreover, the CPDM estimated the entire probability distribution of the data sample. Compared with other generative models devoted to finding the best-fit solution, the CPDM could handle the uncertainty robustly, even if it was trained on a small dataset.

The plentiful and complex features in the sample could exponentially increase the model data requirements. To address the challenges of training with limited medical samples, simplifying the data features was a potential solution. This involved decreasing the number of features and discarding trivial information in the data. In this study, samples used to train the CPDM were grayscale images. Compared with multi-channel color images that contained abundant color information, single-channel grayscale images with only brightness information implied fewer learnable features, which allowed the model to be easier to learn patterns in a small dataset. Furthermore, the sparsity of the medical images made training CPDM on small datasets possible. MRI data utilized in this project are sparse. To be specific, the breast tissues occupied the central area of the projections, and the edges were filled by black pixels with zero or near-zero pixel values. The black edges constituted the sparse regions of data, comprising irrelevant training information. The presence of sparse regions could be regarded as an inartificial feature selection mechanism, which enabled models to focus more on learning a few task-relevant features while ignoring regions containing irrelevant information, to reduce data requirements.

### 4.2. The architecture of the CPDM

Similar to most diffusion model applications in other fields, the CPDM used DDPMs as the basic model framework. The reason DDPMs were widely chosen as the basic framework for diffusion model applications lies in their unique ability to generate high-quality samples, coupled with their broad adaptability to various data types.

To convert genomic information into BC MRIs, conditioning frameworks were incorporated into the DDPM. The common conditioning strategies involve using either concatenation or inner product techniques to fuse multi-modal data from different datasets. In the model architecture design, we initially experimented with concatenation. Drawing from experiences with recurrent neural networks and transformer models, concatenation allowed the model to preserve and utilize information from multiple sources. This approach was crucial for the model to fuse data from different modalities. However, empirical results indicated MRIs synthesized by the model incorporating with concatenation technique only displayed a vague representation of breast tissue, especially when dealing with unpaired datasets. Moreover, when using the different model to generate multiple MRIs for the same patient, there was a significant variance in the content of the images, failing to maintain anatomical consistency. This deviation from the expected uniformity in medical imaging was counterintuitive.

In contrast, the inner product technique provided significant advantages in capturing interactions and correlations between data from two different modalities. Learning the correlations between data from different modalities was crucial for improving the anatomical consistency of synthetic images based on the genomic information of the same person. When different modalities of data (like imaging data and genomic data) were represented as vectors, the inner product could effectively quantify the degree of association between them. This operation resulted in a scalar that encapsulates the joint characteristics of both data modalities, allowing the model to learn from a combined and interaction-driven representation. However, concatenation only simply combined data side by side, aligning features from different modalities without essentially analyzing or understanding their interactions and correlations. Therefore, compared to concatenation, the inner product could convert genomic information into MRI more accurately by capturing the correlations between data from different modalities.

However, there were also some limitations to the inner product technique. Although the model could capture correlations from two different modalities by using the inner product technique, it still lacked a nuanced understanding of context. In visual tasks, this meant the models were possible to miss context-dependent visual contents. The cross-attention mechanism could compensate for this limitation. The cross-attention mechanism allowed the model to focus on relevant parts of genomic data while processing MRIs. This context-aware approach made the synthesized images not only closely align with the genomic information of patients but also exhibited high quality with finer details. The integration of inner product and cross-attention mechanisms in the CPDM capitalized on the strengths of each. This not only elevated the model performance but also improved the visual effects of the synthesized images. The combined method surpassed the capabilities of using either method in isolation.

### 4.3. Analysis for generative model results

In analyzing the performance of CPDM through performance metrics such as FID and MSE, it was observed that although CPDM demonstrated superior performance, the disparity between CPDM and certain baseline models in these metrics was not markedly pronounced. Nonetheless, this should not be construed as indicative of parallel performance between CPDM and baseline models. The FID metric evaluated the similarity in feature space distribution between synthesized and real images, while MSE quantified the average squared discrepancies at the pixel level. A commendable FID score suggested statistical alignment in terms of overall content and style between the generated and actual images, whereas it may not encompass intricacies at the structural or pixel level. Conversely, a small MSE indicated proximity at a pixel resolution but did not inherently assure perceptual congruence. Consequently, though FID and MSE proficiently capture specific facets of MRI quality, they potentially fell short in measuring perceptual and structural congruities. SSIM transcended traditional metrics like MSE, offering a more nuanced and perceptually relevant evaluation. A low SSIM score signified conspicuous deviations in the visual structure between synthetic MRIs and their real counterparts as perceived by human observers. In contrast, a high SSIM denoted high visual information fidelity of synthetic images. Visual fidelity was a critical attribute in medical imaging. Therefore, SSIM elucidated a substantial enhancement in the visual fidelity of MRIs generated by CPDM, distinguishing it from traditional image generation methodologies.

### 4.4. Analysis for application results

In classification-based predictive tasks, the XGBoost model achieved certain success in predicting TP53 gene mutations and ER status based on features extracted from synthetic MRIs. However, as was common in numerous machine learning tasks, due to the imbalanced label distribution in the TP53 mutation status dataset and the ER status dataset, the model had limited generalization capabilities. In contrast, the labels of samples in the ER+/HER2+ dataset were well-proportioned. This harmonious data distribution was a potential drive contributing to the model’s excellent performance.

For the survival analysis based on CPDM-synthesized MRIs, our survival models demonstrated exemplary performance. This success could largely be credited to the CoxPHFilter model. The CoxPHFilter model was proficient at capturing complex, time-dependent patterns in survival data and adeptly managed censored data. Its robust hazard function modeling and adaptability to diverse dataset traits enhanced the accuracy and dependability of prognostic predictions from CPDM-generated MRIs. Essentially, this represented a synergistic blend of advanced imaging synthesis and refined statistical modeling, leading to improved outcomes in clinical research.

Finally, in the classification tasks, the model performed best when trained on MRI features extracted by the Pyradiomics tool. Pyradiomics tool captures a comprehensive range of features, including shape, texture, and intensity, which may be closely associated with the irregular tumor borders and heterogeneity observed in MRIs of patients with TP53 gene mutations, as well as the distinct tumor growth patterns and tissue density found in ER+ patients. For survival analysis, models using features extracted by ResNet50 showed superior performance. ResNet50’s deeper architecture and residual connections enable it to capture more complex and detailed features, which are crucial for understanding intricate patterns associated with disease progression and patient outcomes. While VGG16 and InceptionV3 also contribute valuable features, their architecture may not be as well-suited for the specific demands of our tasks. VGG16, although good at capturing hierarchical features, may lack the specificity needed for medical images. InceptionV3, despite its efficiency in capturing multi-scale features, sometimes results in less focused feature extraction due to its complexity.

## 5. Conclusions

This study has demonstrated the training process of CPDM. The empirical results show the strong potential of CPDM in medical image synthesis. The repeated experiments on the gene expression dataset indicate the wide adaptability of CPDM. Moreover, the application results discern that the synthetic MRIs can be utilized to train the models that are used to predict clinical attributes in the real world. In the future, we aim to develop more AI techniques to achieve targeted treatment of BC, based on specific gene mutations and molecular characteristics associated with various BC subtypes identified in this study. This approach is envisaged to yield more precise, effective, and personalized treatment methods, ultimately enhancing patient outcomes and impacting the field of cancer research more broadly.

## Supporting information

S1 Fig Loss diagram of CPDMs. **A.** multi-omic version. **B.** gene expression version.
(TIF)

S2 Fig K-means clustering results. **A.** Results of clustering the multi-omic profiles of patients. **B.** Results of clustering the gene expression of patients.
(TIF)

S3 Fig SHAP value plot for ER+/HER2+ classification. The plot was based on the XGBoost model trained on the MRI (gene expression version) features extracted by the PyRadiomics tool. The plot showed some important features including entropy, variance, and others. Specifically, entropy could measure the complexity and heterogeneity of pixel intensities, which was crucial for distinguishing different breast tissues. Variance suggested significant variability within the tissue. By effectively utilizing these key aspects of the image data, the model could properly identify subgroup information.
(TIF)

S4 Fig Kaplan-Meier plots (the fold with the best p-value) for CoxPHfilter models trained on features extracted by ResNet50. **A.** The training set of all patients version. **B.** The testing set of all patients version. **C.** The training set of the ER+/HER2+ multi-omic version. **D.** The testing set of the ER+/HER2+ multi-omic version. **E.** The training set of the ER+/HER2+ gene expression version. **F.** The testing set of the ER+/HER2+ gene expression version.
(TIF)

S1 Table The number of features extracted from generated MRIs. (XLSX)

S2 Table Hyper parameters tuning for multi-omic version CPDM. (XLSX)

S3 Table Hyper parameters tuning for gene expression version CPDM. (XLSX)

S4 Table Synthetic image analysis results. (XLSX)

S5 Table Synthetic image analysis results. (XLSX)

S6 Table XGBoost model hyper-parameter tuning for TP53 mutation. (XLSX)

S7 Table XGBoost model hyper-parameter tuning for ER status prediction. (XLSX)

S8 Table XGBoost model hyper-parameter tuning for ER+/Her2+ subtype prediction. (XLSX)

S9 Table Hyper-parameter tuning for multi-omic version (740 patients) CoxPHfilter model. (XLSX)

S10 Table Hyper-parameter tuning for gene expression version (66 patients) CoxPHfilter model. (XLSX)

S11 Table Baseline performance of the classification tasks. (XLSX)

S12 Table Baseline AUPRC cutoff. (XLSX)

S13 Table Baseline performance of the survival analysis. (XLSX)
