# Supplementary figures and images for: Conditional probabilistic diffusion model driven synthetic radiogenomic applications in breast cancer
Source: PLoS Comput Biol. 2024 Oct 7;20(10):e1012490. doi: 10.1371/journal.pcbi.1012490 (PMC11486376; doi:10.1371/journal.pcbi.1012490)

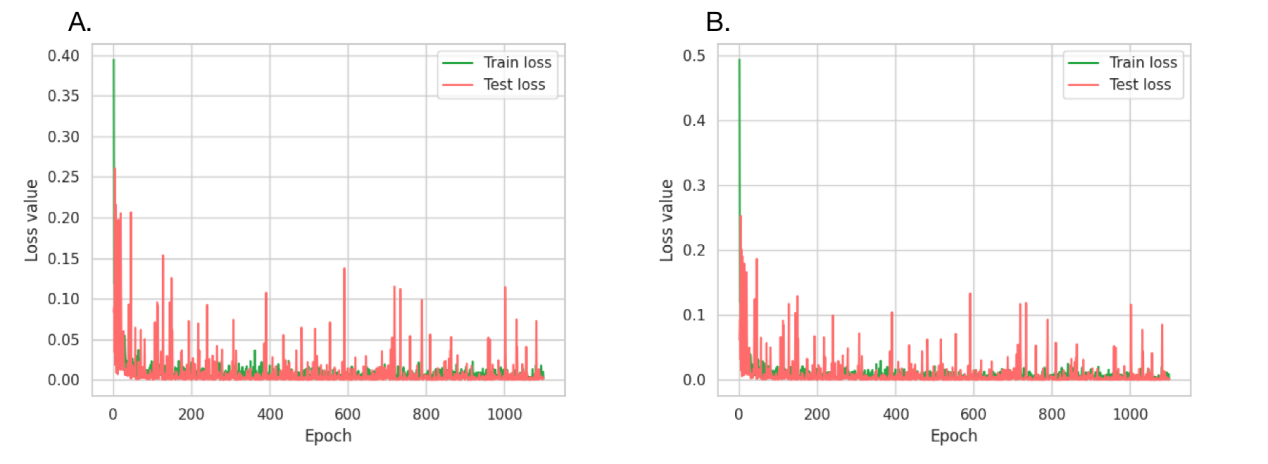

Supplement: S1 Fig — A. multi-omic version. B. gene expression version. (TIF) [file pcbi.1012490.s001.tif]

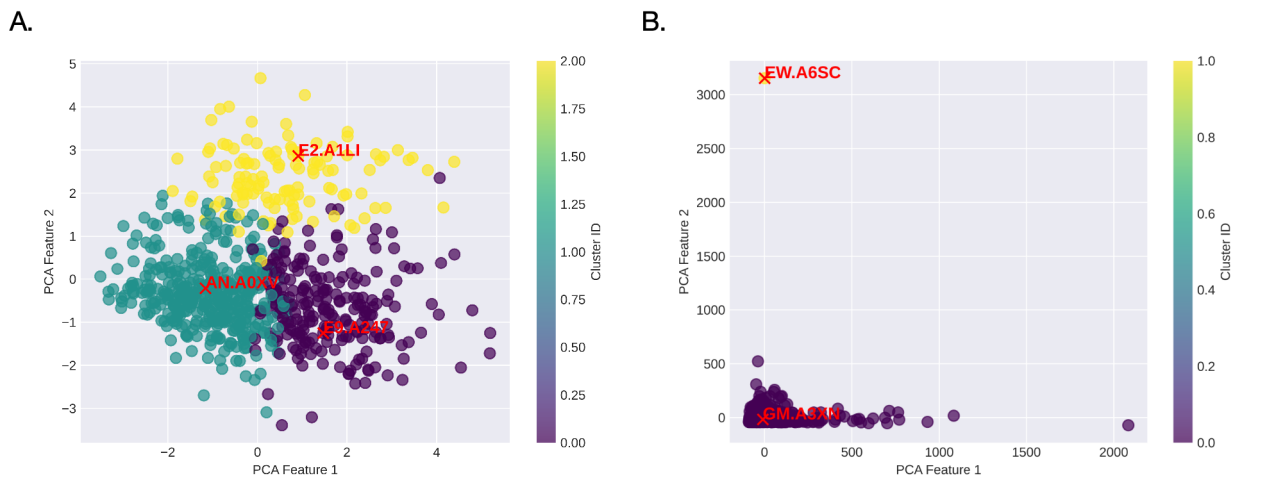

Supplement: S2 Fig — A. Results of clustering the multi-omic profiles of patients. B. Results of clustering the gene expression of patients. (TIF) [file pcbi.1012490.s002.tif]

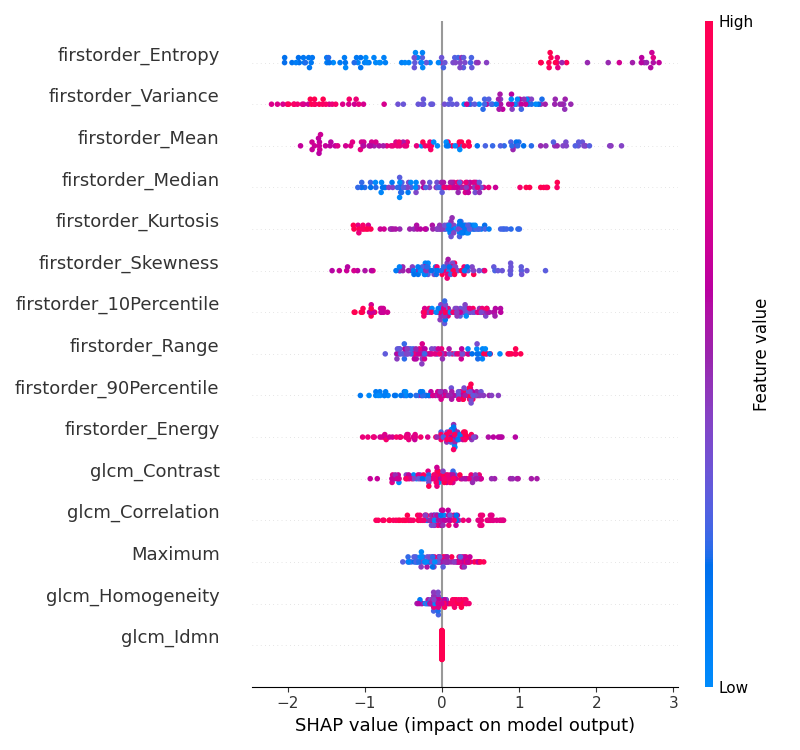

Supplement: S3 Fig — The plot was based on the XGBoost model trained on the MRI (gene expression version) features extracted by the PyRadiomics tool. The plot showed some important features including entropy, variance, and others. Specifically, entropy could measure the complexity and heterogeneity of pixel intensities, which was crucial for distinguishing different breast tissues. Variance suggested significant variability within the tissue. By effectively utilizing these key aspects of the image data, the model could properly identify subgroup information. (TIF) [file pcbi.1012490.s003.tif]

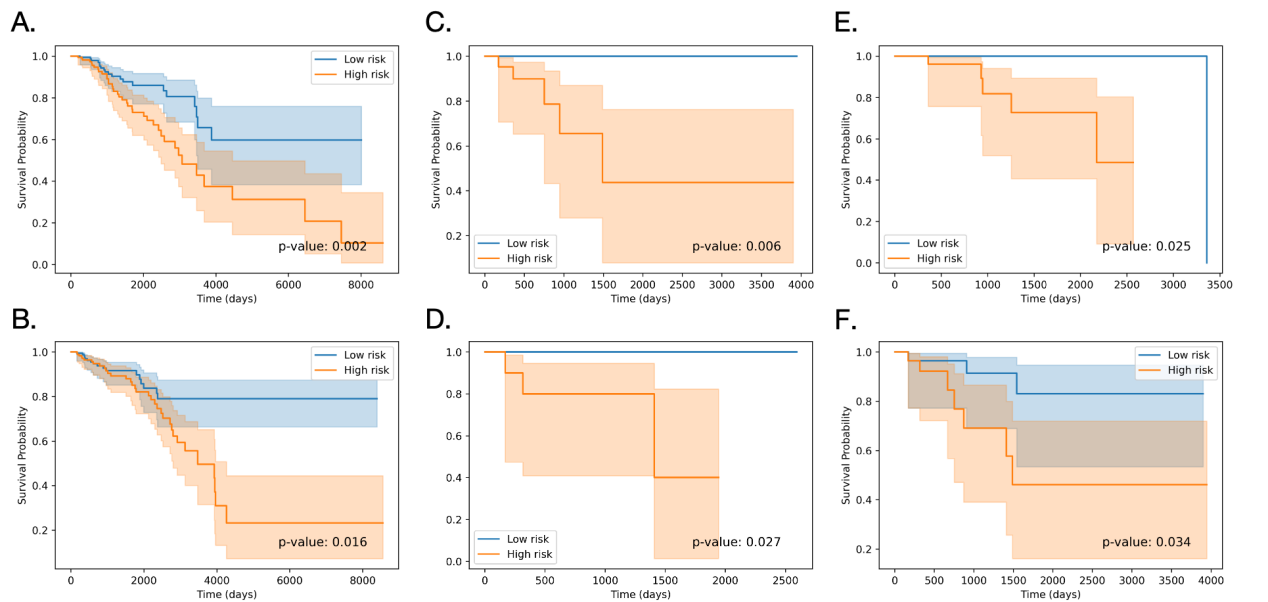

Supplement: S4 Fig — A. The training set of all patients version. B. The testing set of all patients version. C. The training set of the ER+/HER2+ multi-omic version. D. The testing set of the ER+/HER2+ multi-omic version. E. The training set of the ER+/HER2+ gene expression version. F. The testing set of the ER+/HER2+ gene expression version. (TIF) [file pcbi.1012490.s004.tif]
